# Validation of a new wearable device for type 3 sleep test without flowmeter

**DOI:** 10.1371/journal.pone.0249470

**Published:** 2021-04-16

**Authors:** Mauro Contini, Antonio Sarmento, Paola Gugliandolo, Alessandra Leonardi, Gianluigi Longinotti-Buitoni, Camilla Minella, Carlo Vignati, Massimo Mapelli, Andrea Aliverti, Piergiuseppe Agostoni

**Affiliations:** 1 Centro Cardiologico Monzino, IRCCS, Milano, Italy; 2 Dipartimento di Elettronica, Informazione e Bioingegneria, Politecnico di Milano, Milano, Italy; 3 L.I.F.E. Corporation S.A., Luxembourg, Luxembourg; 4 L.I.F.E. Italia S.r.l., Milano, Italy; 5 Department of Clinical Sciences and Community Health, Cardiovascular Section, University of Milano, Milano, Italy; Harper University Hospital, UNITED STATES

## Abstract

**Background:**

Ventilation monitoring during sleep is performed by sleep test instrumentation that is uncomfortable for the patients due to the presence of the flowmeter. The objective of this study was to evaluate if an innovative type 3 wearable system, the X10X and X10Y, is able to correctly detect events of apnea and hypopnea and to classify the severity of sleep apnea without the use of a flowmeter.

**Methods:**

40 patients with sleep disordered breathing were analyzed by continuous and simultaneous recording of X10X and X10Y and another certified type 3 system, SOMNOtouch, used for comparison. Evaluation was performed in terms of quality of respiratory signals (scores from 1, lowest, to 5, highest), duration and classification of apneas, as well as identification and duration of hypopneas.

**Results:**

580 periods were evaluated. Mean quality assigned score was 3.37±1.42 and 3.25±1.35 for X10X and X10Y and SOMNOtouch, respectively. The agreement between the two systems was evaluated with grades 4 and 5 in 383 out of 580 cases. A high correlation (r^2^ = 0.921; p<0.001) was found between the AHI indexes obtained from the two systems. X10X and X10Y devices were able to correctly classify 72.3% of the obstructive apneas, 81% of the central apneas, 61.3% of the hypopneas, and 64.6% of the mixed apneas when compared to SOMNOtouch device.

**Conclusion:**

The X10X and X10Y devices are able to provide a correct grading of sleep respiratory disorders without the need of a nasal cannula for respiratory flow measurement and can be considered as a type 3 sleep test device for screening tests.

## Introduction

There is growing evidence regarding the need for ventilation monitoring during sleep [1–5]. Indeed the presence of sleep apnea is associated with a poor prognosis as well as therapy failure in several diseases such as neurological disorders, heart failure, cardiac arrhythmias, and hypertension [6–11]. Most importantly, sudden death has also been associated with nocturnal hypoxia [12,13]. While monitoring ventilation during sleep, it is important to assess the total number of apneas and hypopneas including their duration, and to distinguish the central, obstructive and mixed apneas according to their varying causes and treatments [14,15]. Finally, ventilation monitoring during sleep is currently performed with various types of sleep tests which, above type 4 (monitoring of oxygen-hemoglobin saturation), implies a relatively uncomfortable experience for the patients considering the complex instrumentation used to measure ventilation [16,17].

Abnormalities of daytime ventilation, either at rest or during exercise, have also been reported albeit studied to a much lesser extent. Indeed, the ability to monitor ventilation during the daytime is limited and specifically, it is difficult to conceive prolonged ventilation measurements through any sort of flow meter during daytime activity [18–20]. Accordingly, there is a need for comfortable and unobtrusive ventilation recording devices capable of quantifying and characterizing ventilation abnormalities, for both day and night [21,22].

In a recent study from our group, we evaluated an innovative wearable device for ECG and respiratory Holter monitoring, which allows noninvasive, continuous, simultaneous, prolonged and accurate monitoring of cardiorespiratory signals [23]. However, no direct comparison with commercially available ventilation recording systems has been done yet. The previous version of this sensorized garment has been upgraded to improve respiratory recording by implementing circumferential sensors instead of linear and local strain gauges. Both ECG and respiratory sensors are woven into the fabric of the self-wearable, and easily washable, fitted garment. The current study was aimed to simultaneously record and compare one standard type 3 system, allowing respiratory movement and airflow measurements through a nasal pressure cannula, with a proposed innovative wearable type 3 system based on respiratory movement measurements only, deriving ventilation from three respiratory sensors embedded in its fabric. More specifically, we evaluated the detection, duration, and classification of apneas, as well as the identification and duration of hypopneas.

## Methods

This is a cross-sectional study conducted in accordance with the guidelines of the Declaration of Helsinki and approved by the ethics committee of the Centro Cardiologico Monzino, IRCCS, Milan, Italy (CCM589). Prior to participating in the study, all patients gave written, signed, and informed consent.

Study inclusion criteria were: age 18–90 years, presence of cardiovascular disease requiring hospitalization and previous diagnosis of moderate to severe sleep apnea/hypopnea assessed by standard polysomnography. Patients using continuous oxygen therapy or with neuromuscular diseases were excluded. All subjects who had access to the Centro Cardiologico IRCCS severe heart failure outpatient unit (between May 2018 and January 2019) and meet the study inclusion/exclusion criteria were asked to participate to the present study.

### Measurements

**Fig 1:**
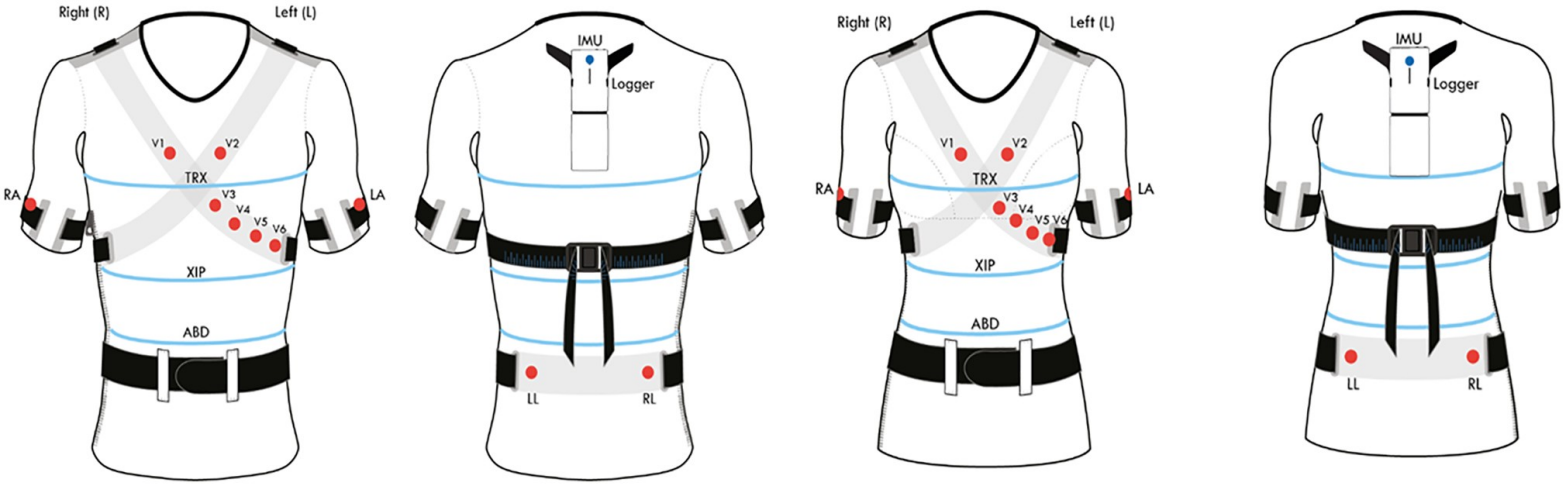
Schematic diagram of X10Y (version for male subjects: Two left images) and X10X (version for female subjects: Two images on the right) medical devices. The garment embeds ten ink-based dry electrodes (red points) to provide 12 lead ECG and three respiratory strain sensors positioned circumferentially around the body at thoracic level (level of the manubrium, TRX), xiphoid process level (XIP), and abdominal level (between the lower costal margin and the umbilical level, AB).

X10X and X10Y medical devices (L.I.F.E. Italia s.r.l., Milano, Italy) is composed of 10 ink-based dry electrodes to monitor ECG, three respiratory strain sensors, one inertial measurement unit (IMU) and a pulse oximeter (Nonin Medical, Minnesota, USA) (Fig 1). For this study, only the respiratory sensors and the oximeter data were analyzed. The respiratory sensors are made of a conductive rubber completely embedded in the garment and positioned circumferentially around the body at thoracic level (level of the manubrium), xiphoid process level, and abdominal level (between the lower costal margin and the umbilical level) (Fig 1). The sensors provide electrical resistance variations measured at a sampling rate of 50 Hz. The devices tested in this study were X10X (version for female subjects) and X10Y (version for male subjects). Both versions have the same number of sensors, hardware and firmware, however, they are characterized by sartorial differences driven by anatomical and ergonomic differences. The female device, in particular, has a specific design enhancing breast support obtained by a light sartorial structure incorporating the cups designed to optimise the contact of both ECG and thoracic respiration circumferential sensors to the skin, thus securing data accuracy even during daily activities/movements. Data of X10X and X10Y devices were analyzed exactly in the same way. Thereafter we refer to X10X-Y device.

The SOMNOtouch^™^ RESP device (Somno SOMNOmedics GmbH, Randersacker, Germany) is composed of a nasal cannula, a pulse oximeter, two respiratory sensors that are positioned at the level of the manubrium and abdomen, and three thoracic electrodes for ECG recording.

### Study protocol

The protocol consisted of a continuous and simultaneous recording of traces from the two devices. All patients wore the SOMNOtouchTM RESP bands and the X10X-Y device in the laboratory at Monzino Hospital where they received practical instructions and where the functioning of the devices was tested by expert medical personnel. After the patients wore the X10X-Y device, signal quality was checked. Thereafter, the SOMNOtouchTM RESP bands were positioned onto the X10X-Y device and all signals were checked again. Only when both devices were judged to provide good quality signals, patients were sent to their home. All recruited patients wore the two devices (X10X-Y and SOMNOtouch^™^ RESP) in the early afternoon, but data acquisition, of both devices was therefore simultaneous and began according to the patients’ sleep habits (usually around 10 pm). The SOMNOtouch^™^ RESP nasal cannula and the finger probes of both instruments were checked, and patients were instructed to wear these devices by themselves just before going to sleep.

Acquisitions started automatically at the set time and lasted all night long: SOMNOtouch^™^ RESP acquisitions ended at the pre-configured time, previously agreed with the patient, while X10X-Yacquisitions were stopped manually before the removal of the garment in the morning.

X10X and X10Y and pulse-oximetry raw data were downloaded from the logger and the watch via USB cables, respectively, stored into a repository on a PC and successively analyzed. Both SOMNOtouch^™^ RESP and X10X-Y data were processed following the standard procedure provided by dedicated software in order to detect obstructive, central and mixed apneas and hypopneas events, and two expert operators successively validated the results produced by automatic analysis.

### Data analysis

Analysis was divided into two parts. The first analysis was a qualitative one. Respiratory signals from SOMNOtouch^™^ RESP and X10X-Y devices were synchronized on the basis of the two on-board clocks. Data regarding respiratory sum signal of both devices (sum of three signals in the X10X-Y devices, and of two signals in the SOMNOtouch^™^ RESP device) were analyzed qualitatively by two experts, and in the case of different scores, they were averaged. More specifically, every 30 minutes, an interval of 8 minutes was analyzed by giving grades that ranged from 1 to 5, i.e. 1 = poor signal and difficult interpretation; 2 = insufficient signal and questionable interpretation; 3 = sufficient signal and reliable interpretation; 4 = good signal and reliable interpretation; 5 = excellent signal and reliable interpretation. An analogous 1 to 5 grading was carried out in order to describe the level of agreement between the quality of SOMNOtouch^™^ RESP and X10X-Y respiratory sum signals. A set of figures reporting examples of grade 1 to 5 is reported in the supplemental file material as S1–S5 Figs.

The second analysis, also performed by two expert operators, was a quantitative one. The data derived from SOMNOtouch^™^ RESP were automatically processed by SOMNOMedics software for detection of sleep respiratory disorders. Respiratory event scoring was then manually confirmed according to the most recent guidelines [24], considering the signals of the thoracoabdominal bands, the flowmeter and the pulse-oximeter. The data derived from X10X-Y devices, after filtering them with a pass band filter 0.045–1 Hz, were processed in a similar way but only using the three thoracoabdominal bands together with their sum and oxygen saturation signal. Similarly, the first step was an automatic apneas and hypopneas (‘events’) detection by X10X-Y software, based on the following criteria definitions:

- event: minimum duration of 10 seconds and ≥3% oxygen desaturation [24];
- hypopnea: signal excursion drop by ≥30% with respect to the previous minute;
- central apnea: signal excursion drop by ≥80% with respect the previous minute;
- obstructive apnea: signal excursion drop by ≥50% with respect to the previous minute, in presence of thoraco-abdominal asynchrony (phase angle > 36 degrees);
- mixed apnea: signal excursion drop by ≥50% with respect to the previous minute, with an initial phase without efforts followed by a second phase with efforts (presence of thoraco-abdominal asynchrony).

After automatic analysis performed by the software, respiratory event scoring was successively manually confirmed according to the most recent guidelines [24]. In detail, the criteria used for hypopneas were the ones classified in [24] as 1A (“Scoring of Hypopneas” section), namely: a) the peak signal excursion drop by ≥30% of pre-event baseline using an alternative hypopnea sensor; b) the duration of the ≥30% drop in signal excursion is ≥10 seconds; c) there is a ≥3% oxygen desaturation from pre-event baseline or the event is associated with an arousal.

**Fig 2:**
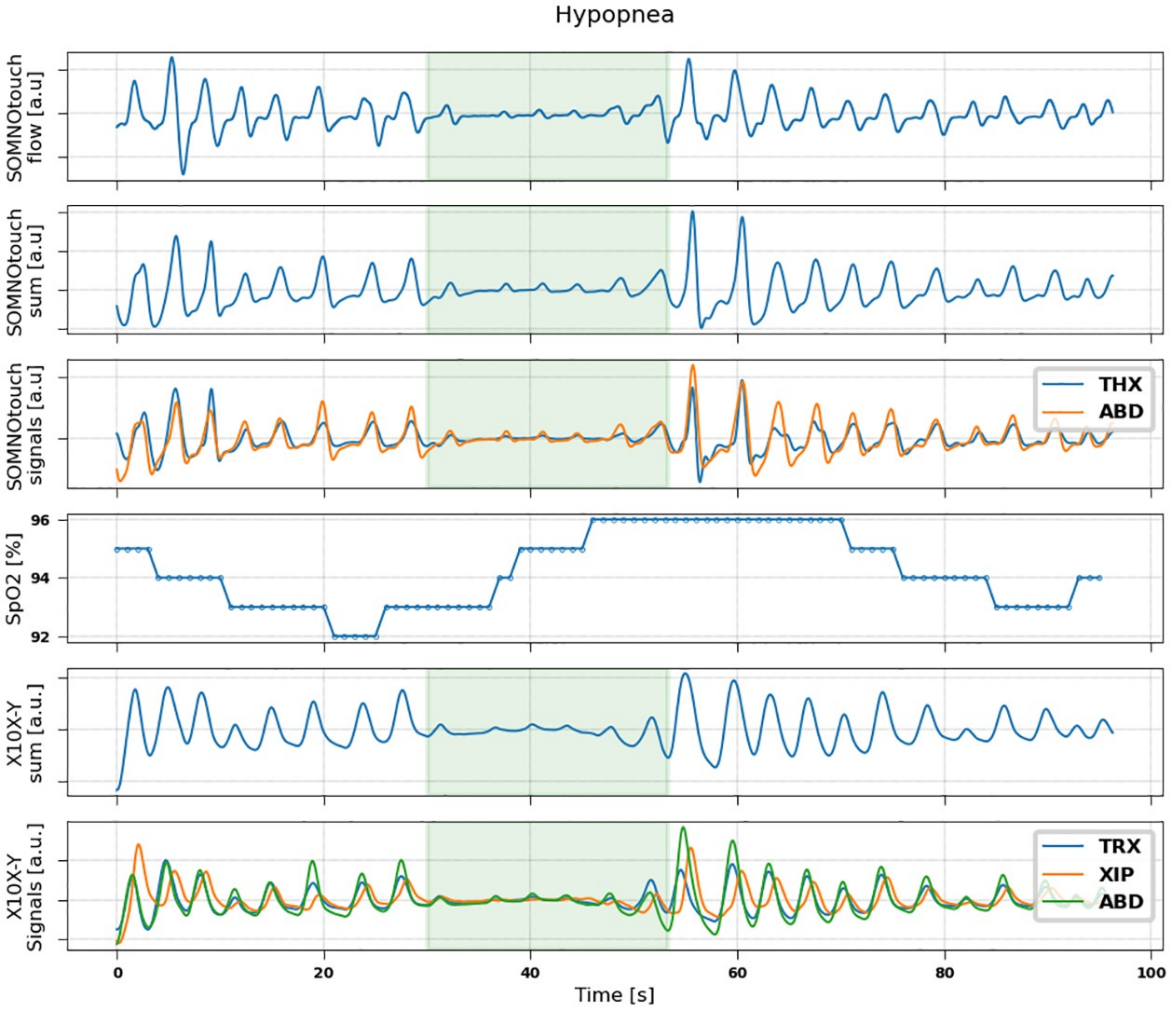
Representative example of signals measured during a hypopnea event in a patient (79 years, male, BMI = 23.3). From top to bottom: flow (measured by SOMNOtouch device); sum signal (thoracic + abdominal) obtained from SOMNOtouch device; thoracic (blue) and abdominal (orange) signals from SOMNOtouch device; oxygen saturation from pulse oximeter; sum signal (thoracic +xiphoid + abdominal) obtained from X10X-Y device; thoracic (blue), xiphoid (orange) and abdominal (green) signals from X10X-Y device.

**Fig 3:**
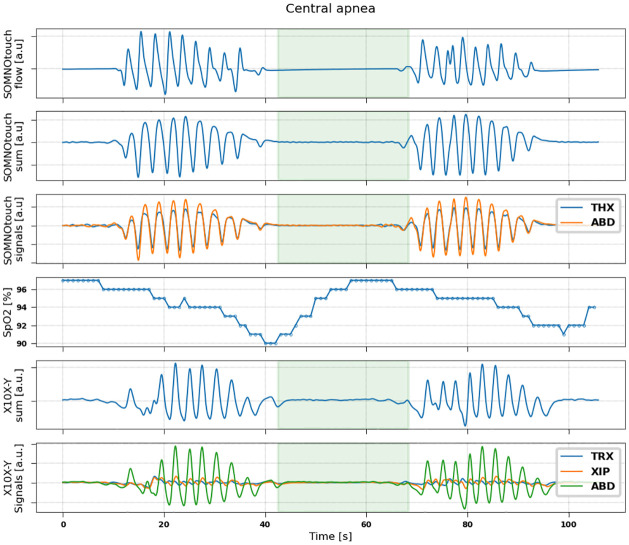
Representative example of signals measured during a central apnoea event in a patient (71 years, male, BMI = 26.2). From top to bottom: flow (measured by SOMNOtouch device); sum signal (thoracic + abdominal) obtained from SOMNOtouch device; thoracic (blue) and abdominal (orange) signals from SOMNOtouch device; oxygen saturation from pulse oximeter; sum signal (thoracic +xiphoid + abdominal) obtained from X10X-Y device; thoracic (blue), xiphoid (orange) and abdominal (green) signals from X10X-Y device.

**Fig 4:**
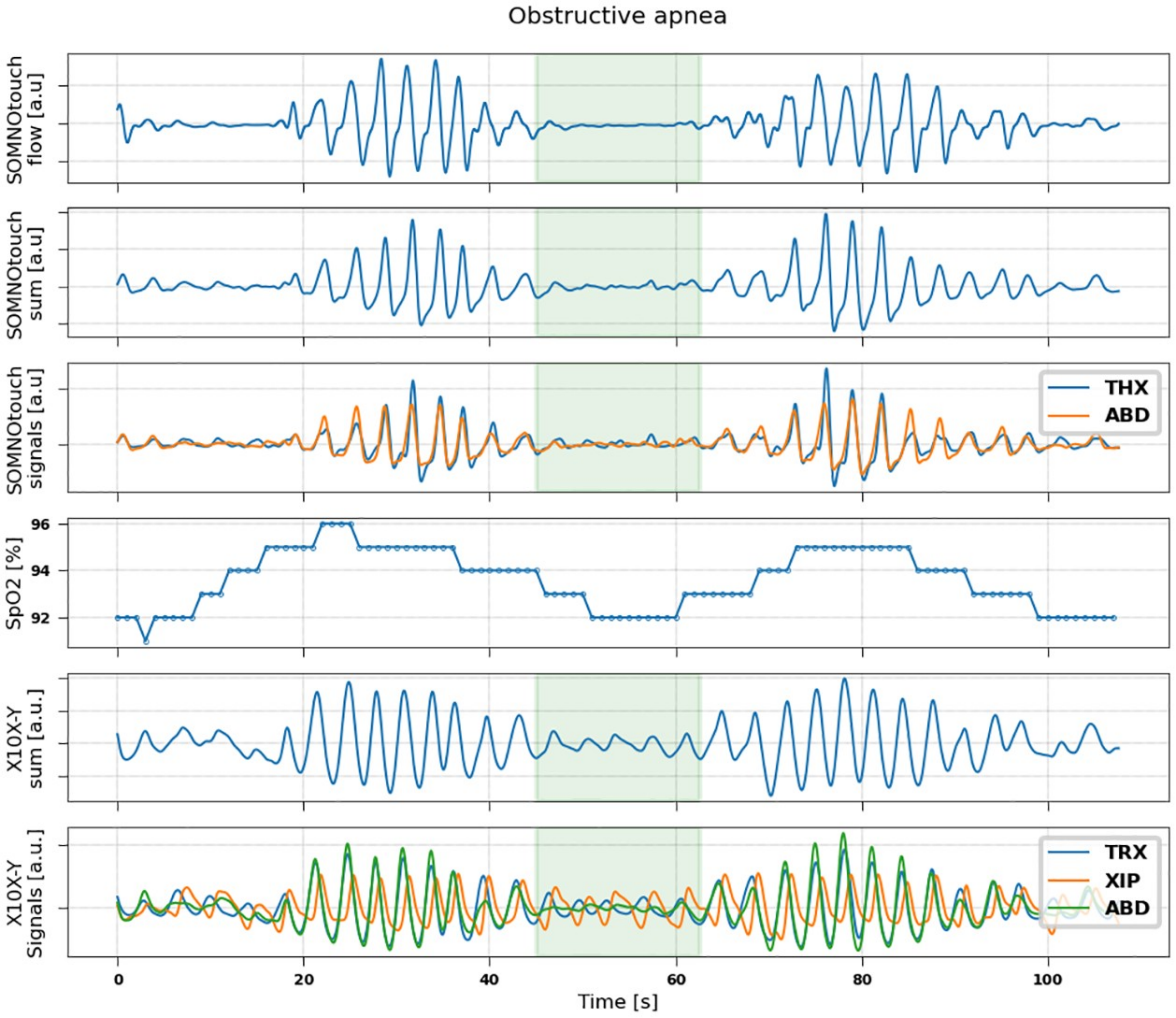
Representative example of signals measured during an obstructive sleep apnoea event in a patient (79 years, male, BMI = 23.3). From top to bottom: flow (measured by SOMNOtouch device); sum signal (thoracic + abdominal) obtained from SOMNOtouch device; thoracic (blue) and abdominal (orange) signals from SOMNOtouch device; oxygen saturation from pulse oximeter; sum signal (thoracic +xiphoid + abdominal) obtained from X10X-Y device; thoracic (blue), xiphoid (orange) and abdominal (green) signals from X10X-Y device.

**Fig 5:**
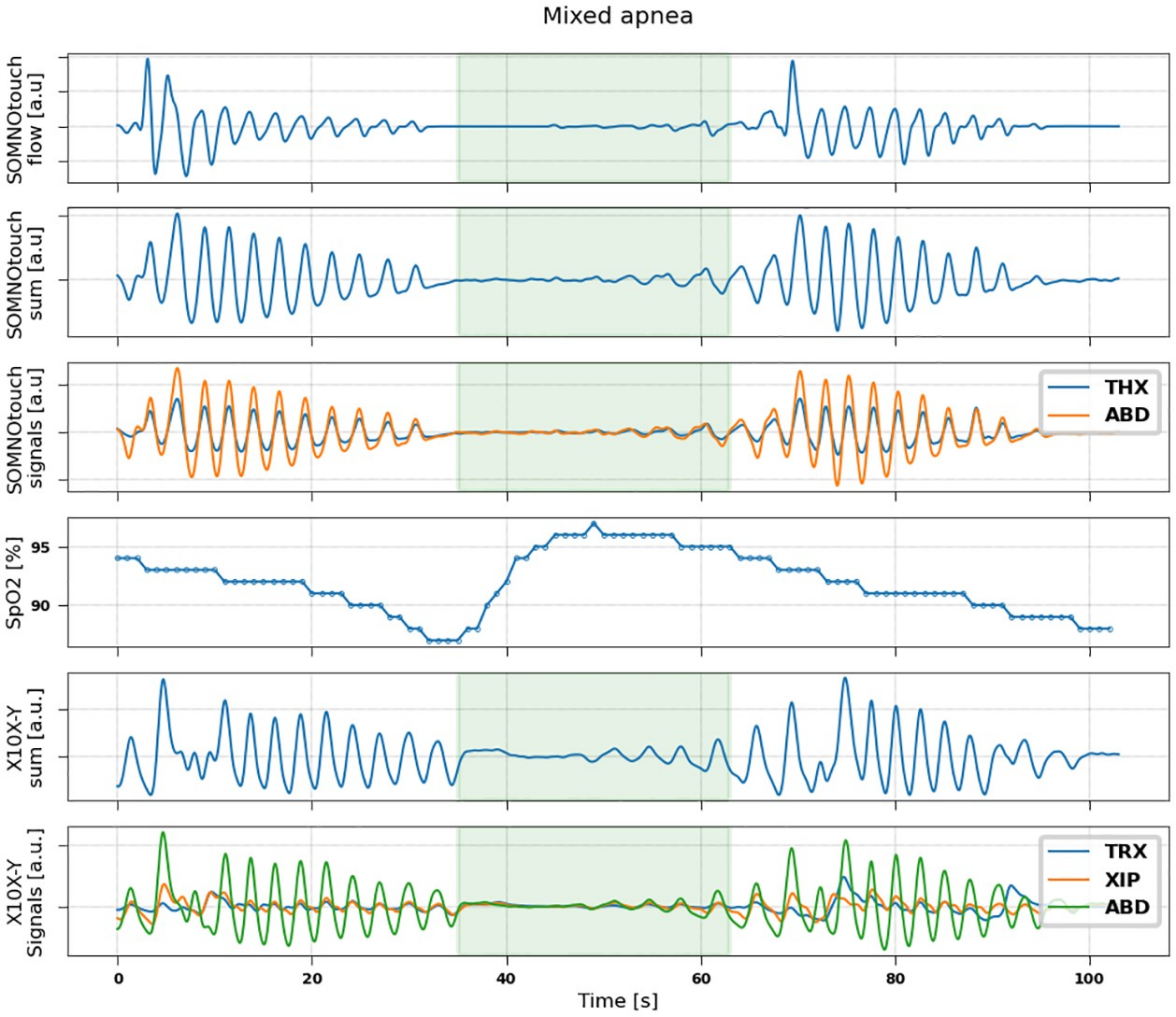
Representative example of signals measured during a mixed apnoea event in a patient (71 years, male, BMI = 26.2). From top to bottom: flow (measured by SOMNOtouch device); sum signal (thoracic + abdominal) obtained from SOMNOtouch device; thoracic (blue) and abdominal (orange) signals from SOMNOtouch device; oxygen saturation from pulse oximeter; sum signal (thoracic +xiphoid + abdominal) obtained from X10X-Y device; thoracic (blue), xiphoid (orange) and abdominal (green) signals from X10X-Y device.

Examples of signals recorded by the two devices during events of hypopnea, central, obstructive and mixed apneas are shown in Figs 2–5.

Apnea-hypopnea index (AHI) was graded as mild (≥5 and <15 events/hour), moderate (≥15 and <30) and severe (≥30) [24]. After the operator’s correction, the set of events detected by SOMNOtouch^™^ RESP and X10X-Y devices were compared and classified as true positive (TP, when both systems detected the same type of event at the same time), false positive (FP, when an event of a specific type detected by the X10X-Y devices, was not detected by the SOMNOtouch^™^ RESP device), and false negative (FN, when an event of a specific type detected by the SOMNOtouch^™^ RESP system, was not detected by the X10X-Y devices).

### Statistical analysis

Data normality was verified using the Shapiro-Wilk test. Metrics of sensitivity (SE) and positive predictivity (+P) were used to summarize the results of comparison as follows:

$$SE=\frac{TP}{TP+FN}$$

$$+P=\frac{TP}{TP+FP}$$

A 5x5 confusion matrix was used to cross-validate the detected events during sleep (i.e. hypopneas, obstructive, central, and mixed apneas) between devices. Agreement regarding AHI between SOMNOtouch^™^ RESP and X10X-Y were evaluated using Bland-Altman plot, and the results were presented as bias with limits of agreement (±1.96 STD). In order to observe if the limits were acceptable, the 95% confidence interval was calculated for both bias and limits of agreement according to Giavarina [25]. Correlations were studied using Pearson correlation coefficient and both coefficients of correlation (r) and determination (r^2^) were calculated.

Data analysis was performed using Python scipy library (submodule stats) version 1.2.1. A p-value of <0.05 (2-sided) was considered statistically significant.

## Results

**Fig 6:**
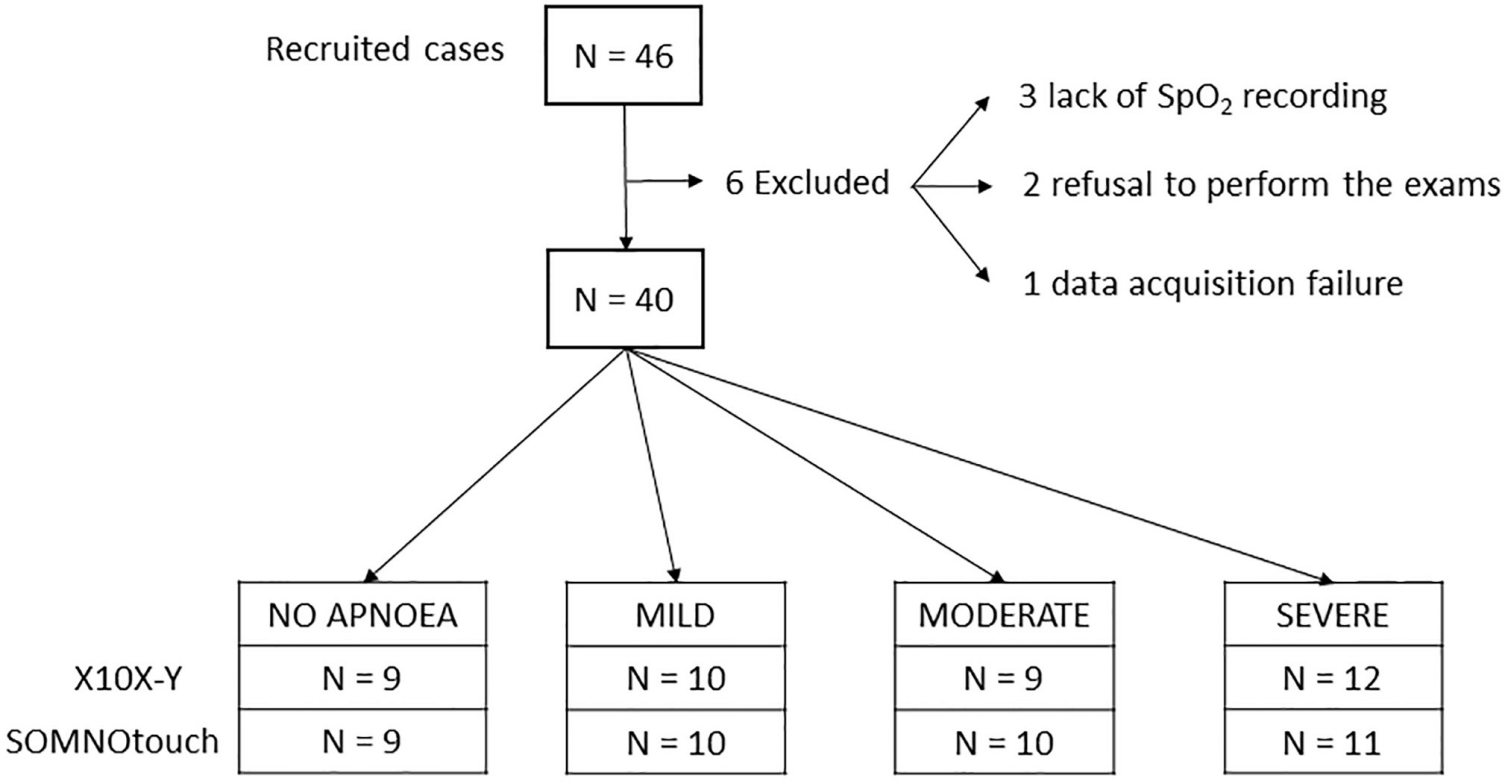
Patient flow and apnea severity as assessed by X10X-Y and SOMNOtouch devices.

Forty-six patients were recruited (42 males, 4 females). Median age was 69 years (STD 11 years, range 39–85 years). Of these, 6 patients were excluded for different reasons: the lack of SpO_2_ recording (3 patients), the refusal to perform the exams (2 patients) and the failure of the acquisition (1 patient) (Fig 6). Demographic and clinical characteristics of the 40 patients who concluded the study are reported in Table 1. Average AHI at the pretest evaluation was 25.77±9.68 which included obstructive, central and mixed apneas (Table 1).

**Table 1:** Demographic and clinical characteristics of studied population.

SEX [M/F]	34/4
AGE [YEARS]	68.7 ± 11
HEIGHT [CM]	172.3 ± 8.2
WEIGHT [KG]	85.8 ± 17.8
BMI [KG/M2]	28.8 ± 5.1
CHEST CIRCUMFERENCE [CM]	108.6 ± 11.6
ABDOMINAL CIRCUMFERENCE [CM]	110.1 ± 14.9
RECORDING DURATION [H]	7.28 ± 0.9
EVENTS [N]	133.12 ± 87.58
AI [APNEA EVENTS/H]	18.58 ± 11.16
AHI [APNEA+HYPOPNEA EVENTS/H]	25.77 ± 9.68
OBSTRUCTIVE APNEAS INDEX [EVENTS/H]	12.17 ± 9.28
CENTRAL APNEAS INDEX [EVENTS/H]	4.1 ± 8.1
MIXED APNEAS INDEX [EVENTS/H]	2.34 ± 4.55
HYPOPNEAS INDEX [EVENTS/H]	49.77 ± 44.86

BMI = body mass index; AI = apnea index (events/hour); AHI = apnea hypopnea index (events/hour).

Polysomnography data refer to pre-test analysis.

Mean quality assigned score was 3.37±1.42 and 3.25±1.35 for X10X-Y and SOMNOtouch^™^ RESP, respectively. Higher values of quality assessment (grades 4 and 5, S4 and S5 Figs, online supplement) were more frequently assigned to X10X-Y than to SOMNOtouch^™^ RESP recordings, while the opposite occurred for lower grades (1 and 2) (S1 and S2 Figs, online supplement). A good agreement, (grades 4 and 5) was observed in most cases (383/580).

**Fig 7:**
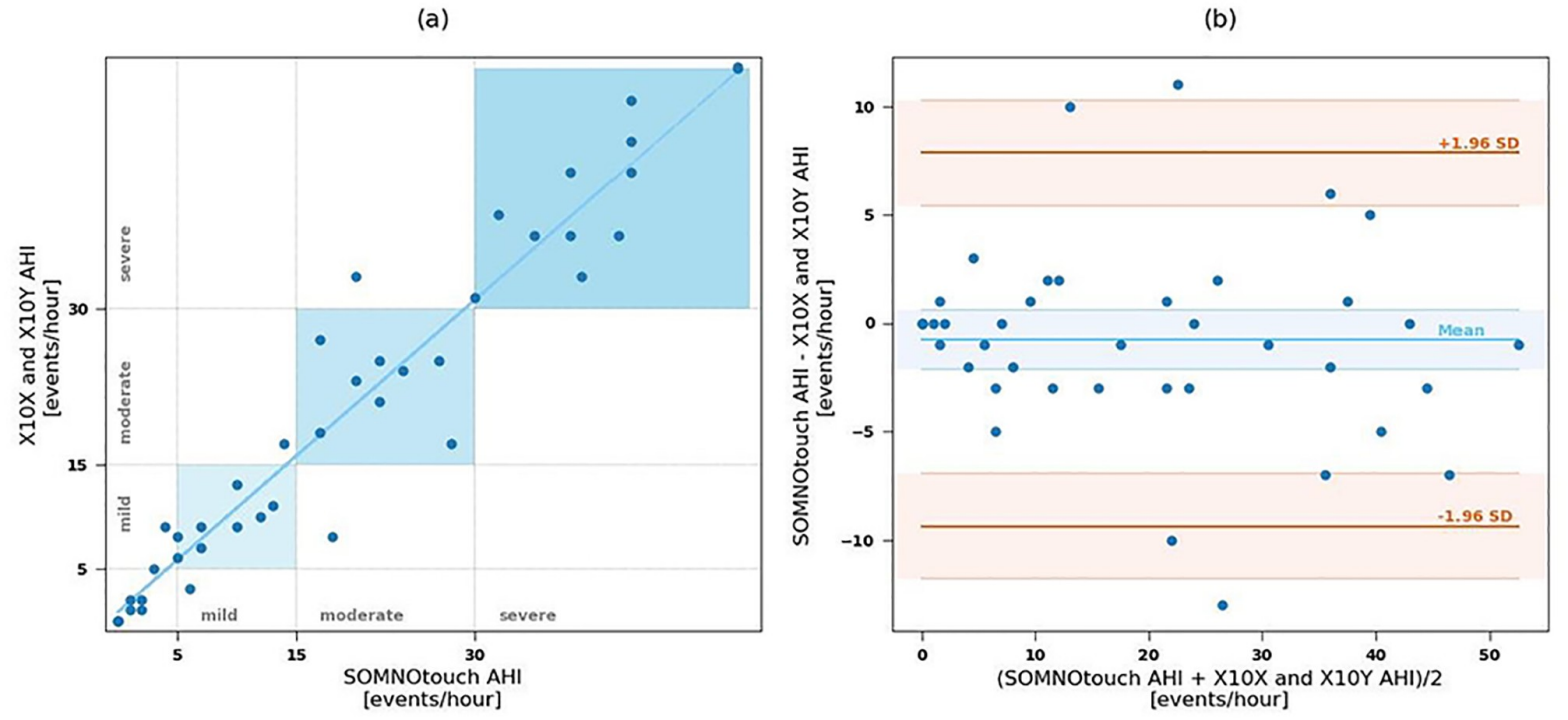
Left panel: Correlation between apnea hypopnea index (AHI) observed with X10X and X10Y and SOMNOtouch^™^ RESP devices. Squares represent areas of agreement in the AHI severity classification. Right Panel: Bland-Altman plot of AHI detected by X10X and X10Y and SOMNOtouch^™^ RESP devices. Confidence intervals of bias and limits of agreement are shown.

All included patients AHI were graded as mild, moderate and severe with X10X-Y in 10, 9, and 12 patients, respectively, while SOMNOtouch^™^ RESP graded the same AHI in 10, 10, and 11 patients (Fig 7). Apnea/hypopnea was not observed in seven cases by both devices. Five out of 38 patients were classified differently by the two devices. Specifically, 3 patients classified as “mild” by X10X-Y, were classified by SOMNOtouch^™^ RESP as “normal” in two cases and “moderate” in one case; two patients classified as “normal” and “moderate” by X10X-Y, were classified as “mild” by the SOMNOtouch^™^ RESP, and two patients classified as “severe” by X10X-Y, were classified as “moderate” by SOMNOtouch^™^ RESP. Nevertheless a high correlation (r = 0.960; r^2^ = 0.921; p<0.001) and a good agreement (mean bias -0.77±4.39) were found between devices.

Average recording duration was 7.4±1.1 h. When considering all events detected by X10X-Y device, the X10X-Ydevice was able to correctly classify 72% of the obstructive apneas, 81% of the central apneas, 61% of the hypopneas, and 64.6% of the mixed apneas when compared to the SOMNOtouch^™^ RESP device (Table 2). When considering all events detected by SOMNOtouch, the X10X-Y device was able to correctly classify 56% of the obstructive apneas, 69% of the central apneas, 38% of the hypopneas, and 56% of the mixed apneas when compared to the SOMNOtouch^™^ RESP device (Table 2).

**Table 2:** The 5x5 confusion matrix computed for classified events between X10X-Y and SOMNO devices.

	**X10X-Y**					
**SOMNO**		**OA**	**CA**	**Hypopnea**	**MA**	**X**
	**OA**	**1485**	276	133	158	596
	**CA**	28	**801**	11	148	164
	**Hypopnea**	164	26	**319**	11	294
	**MA**	99	255	37	**715**	163
	**X**	613	161	625	58	-

The diagonal cells (in black) represent correctly classified events between devices s (number of occurrences in each cells are given as N). Off-diagonal cells represent various events of misclassification. OA: Obstructive apnea; CA: Central apnea; MA: Mixed apnea; X: Unrecognized event.

The SE and +P of X10X-Y in identifying a total number of events was 78% and 76%, respectively (Table 3).

**Table 3:** Total sum of events identified by both devices.

	X10X and X10Y	SOMNOtouch^™^	SE	+P
**OA**	2396	2640	56	61
**CA**	1521	1152	69	52
**Hypopnea**	1127	805	38	28
**MA**	1093	1263	56	65
**TOTAL**	6137	5861	78	76

OA: Obstructive apnea; CA: Central apnea; MA: Mixed apnea; SE: Sensitivity, +P: Positive predictivity.

## Discussion

Our study compared a new wearable device for the simultaneous recording of multiple cardiorespiratory signals (X10X-Y devices) to an approved type 3 recording sleep device, a well-established, and widely used nocturnal cardiorespiratory monitoring system in the clinical field (SOMNOtouch^™^ RESP). We showed that X10X-Y are able to provide during sleep reliable respiratory signals, reliable classification of patients apnea severity and accurate classification of apnea/hypopnea events.

Some operating differences between the two devices need to be acknowledged:

1. X10X-Y devices are based on an easily wearable garment with sensors integrated into the fabric so that their position is reliable and consistent throughout the recording period. Differently, the respiratory bands in the SOMNOtouch^™^ RESP system, as well as other commercial sleep devices, face the possibility of band dislodgment;
2. In the SOMNOtouch^™^ RESP system, respiratory flow is directly recorded by a nasal cannula measuring changes in nasal air pressure during ventilation, which are used to detect and classify sleep events. In X10X-Y devices, conversely, respiratory flow is derived from an integrated analysis of respiratory movements detected at three different levels of the thoraco-abdominal wall;
3. In SOMNOtouch^™^ RESP system, classification of apneas is made by simultaneous analysis of the behavior of respiratory flow and respiratory thoracic and abdominal respiratory movements. This is because a central apnea is detected when both airflow and respiratory movements are absent, and obstructive when airflow is absent in the presence of thoracic and/or abdominal respiratory movement. Conversely, in the X10X-Y devices, both central and obstructive apneas are characterized by a flattening of the sum signal of respiratory movement traces, but an obstructive event is recognized if abdominal and thoracic movements are out of phase (S3 Fig, online supplement). This is the reason why in our X10X-Y device, we have differentiated the threshold used to detect central apneas and obstructive apneas in order to be as sensitive as possible, i.e. to detect the highest possible number of events when characterizing a patients in terms of AHI.

The qualitative analysis showed a slightly higher quality of the X10X-Y respiratory sum signal in comparison to the one recorded by the SOMNOtouch^™^ RESP system. The sensors embedded in X10X-Y fitted garment are positioned more firmly, and this likely account for the difference.

As for quantitative analysis, despite the reported technical differences in the system operating features, a good correlation has been found in AHI determination between the two devices. Moreover, the severity grading for respiratory disorders showed a very high agreement between the SOMNOtouch^™^ RESP and X10X-Y devices. Indeed, in only 5 patients out of 40 (12.5%) severity disease estimation differed between the two devices and in all cases, the difference was of only one class of severity. Notably in all but one patient with severe apnea/hypopnea as well as in all patients with no sleep abnormalities X10X-Y properly detected the sleep severity of the sleep disorder.

It is noteworthy that the correct detection of sleep apneas/hypopneas severity with X10X-Y devices, as validated by agreement with SOMNOtouch^™^ RESP event detection, was obtained in the absence of a direct respiratory airflow recorded by a nasal cannula [16,18,22]. Avoiding the application of a nasal cannula is highly favorable from several points of view: higher patient comfort, (i.e. reduced interference with sleep quality induced by the monitoring system), lower risk of signal loss, and reduced possibility of apneas under-detection in case of prevalent mouth respiration. Moreover, the absence of a nasal cannula is more consistent with the use of the system for a prolonged time, as it could happen for a continuous nighttime and daytime recording [26,27].

We must recognize, however, that the agreement between the two devices drops if respiratory event classification is taken into account. A good accuracy is still present for central and obstructive apneas, but both sensitivity and positive predictive values fall below 50% for hypopneas. This is not surprising if the operating differences between the two recording systems is considered, as the absence of direct respiratory flow measurement becomes, in this case, a major limitation. Indeed, on the basis of the only respiratory movement analysis, discrimination between an obstructive apnea and a hypopnea substantially depends on the presence of thoraco-abdominal asynchrony, because events with a counter-phase angle close to the cut off value could be easily misdiagnosed.

A few study limitations should be acknowledged. Firstly, patients comfort scale while wearing X10X-Y and SOMNOtouch^™^ RESP were not assessed in the present study. This is due to the simultaneous application of the two systems so that comfort analysis for each device was not possible. Secondly, daily respiratory recordings were not assessed because SOMNOtouch^™^ RESP is not designed for daily ambulatory monitoring. However in a recent study we analyzed X10X-Y devices continuously for 24 hours in 10 healthy subjects and 30 cardio respiratory patients and observed promising findings [23]. Thirdly, our patients population was unbalanced toward male subjects as expected for a study analazing consecutive patients with sleep disorders. Therefore future studies dedicated to separately assess the capability of X10X and X10Y devices in females and males are needed. Finally, while the quantitative analysis was performed over the entire data recording, the qualitative analysis was performed by visual inspection of 8 minutes samples taken every 30 minutes, choosing intervals in a blind fashion and using a predetermined fixed interval.

In conclusion, the X10X and X10Y devices are a reliable, non-invasive device for recording sleep respiratory behavior, able to provide a correct grading of sleep respiratory disorders when compared to a widely used system for nocturnal cardiorespiratory monitoring like SOMNOtouch^™^ RESP, even in the absence of direct measurement of the respiratory flow. At present, the X10X and X10Y devices can be considered as a type 3 sleep test device but it should be principally regarded as a screening test for the detection and quantification of sleep respiratory disorders, as well as a daily monitoring system for early detection, to reveal information that would have otherwise been overlooked. On the other hand, the unrequired need of a nasal cannula for respiratory flow measurement presents an excellent step forward for respiratory analysis during the daytime, and for everyday activities.

## Supporting information

S1 Fig Example of grade 1 = poor signal and difficult interpretation. (DOCX)

S2 Fig Example of grade 2 = insufficient signal and questionable interpretation. (DOCX)

S3 Fig Example of grade 3 = sufficient signal and reliable interpretation. (DOCX)

S4 Fig Example of grade 4 = good signal and reliable interpretation. (DOCX)

S5 Fig Example of grade 5 = excellent signal and reliable interpretation. (DOCX)
