# Supplementary figures and images for: Validation of a new wearable device for type 3 sleep test without flowmeter
Source: PLoS One. 2021 Apr 16;16(4):e0249470. doi: 10.1371/journal.pone.0249470 (PMC8051765; doi:10.1371/journal.pone.0249470)

**Figure 1s.**


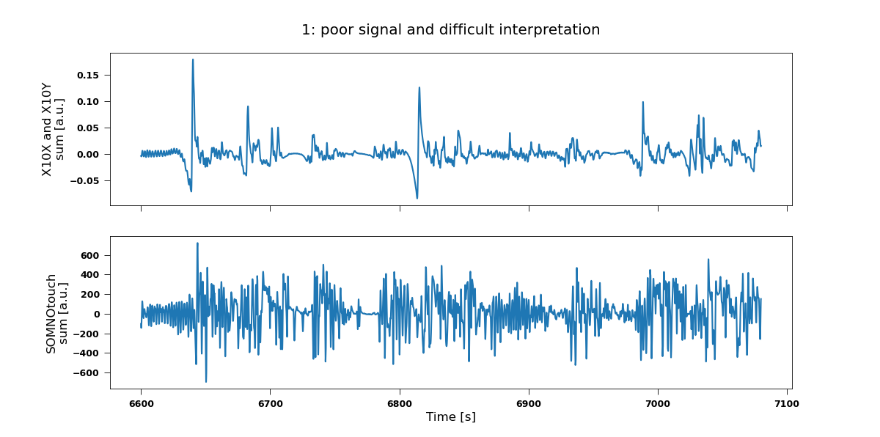


Example of grade 1 = poor signal and difficult interpretation

Supplement: S1 Fig — (DOCX) [file pone.0249470.s001.docx]

**Figure 2s.**


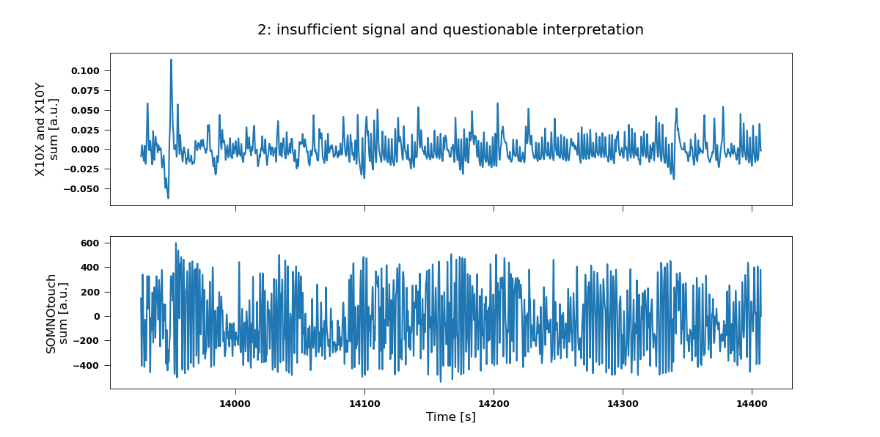


Example of grade 2 = insufficient signal and questionable interpretation

Supplement: S2 Fig — (DOCX) [file pone.0249470.s002.docx]

**Figure 3s.**


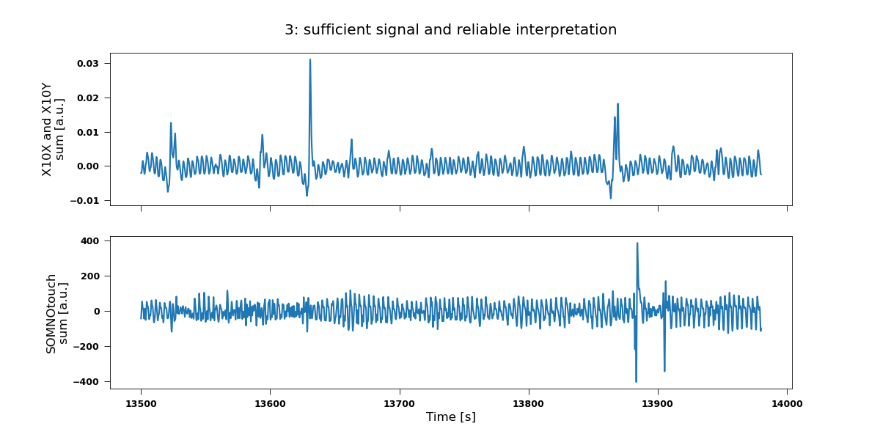


Example of grade 3 = sufficient signal and reliable interpretation

Supplement: S3 Fig — (DOCX) [file pone.0249470.s003.docx]

**Figure 4s.**


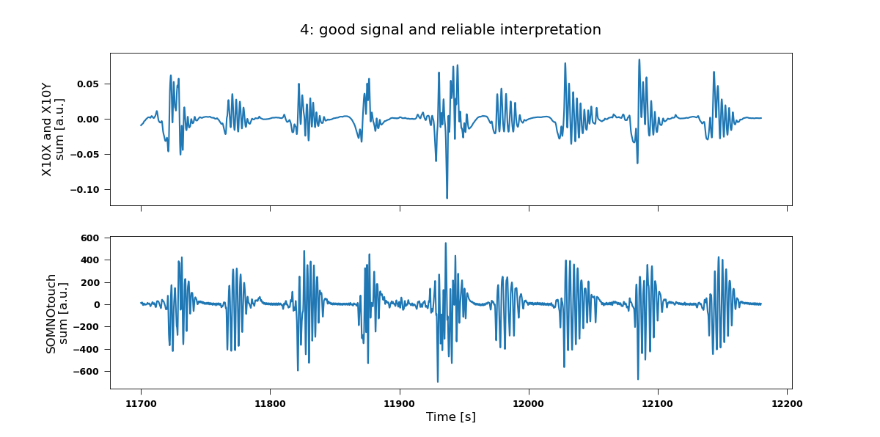


Example of grade 4 = good signal and reliable interpretation

Supplement: S4 Fig — (DOCX) [file pone.0249470.s004.docx]

**Figure 5s.**


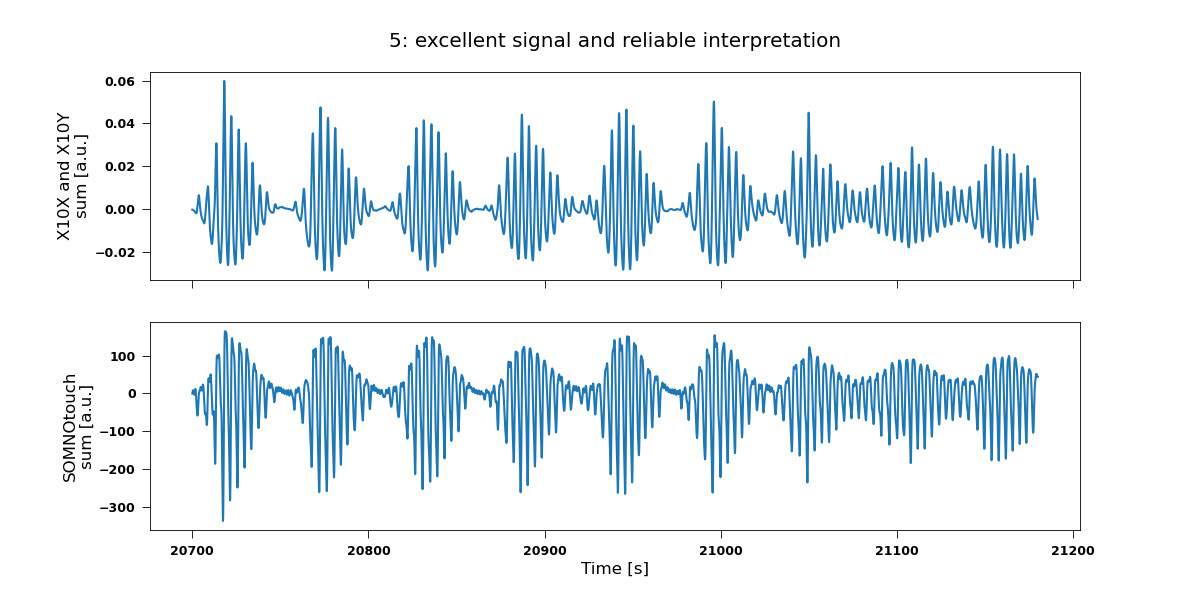


Example of grade 5 = excellent signal and reliable interpretation

Supplement: S5 Fig — (DOCX) [file pone.0249470.s005.docx]
